# SPEED IN GERIATRICS EDUCATION: IDENTIFYING AND IMPLEMENTING MICROLEARNING TO INCREASE EFFICIENCY AND RETENTION

**DOI:** 10.1093/geroni/igae098.1084

**Published:** 2024-12-31

**Authors:** Evan Henricks, Pratima Gangupantula

**Affiliations:** Medical College of Wisconsin, Milwaukee, Wisconsin, United States; The University of Texas Health Science Center San Antonio, San Antonio, Texas, United States

## Abstract

This session aims to explore what microlearning is and how it can be utilized by geriatrics educators to reinforce and incorporate age-friendly health elements across the spectrum of learners. Microlearning is obtaining knowledge or skills via small units and is increasingly endorsed by health professions educators as a means of facilitating learning, training, and continuing education. The positive impact of microlearning on learner knowledge and confidence has been shown. Microlearning can be integrated with mobile learning (M-Learning) to create learning material that is quick, interactive, and easily accessible from a mobile device. This is in contrast to electronic learning (E-learning) which allows online access to traditional resources like textbooks, journals, and lectures, but is in-depth, cumbersome, and may lack open access. This session will explore examples of situations which utilize various microlearning modalities for geriatrics education, such as social media platforms, podcasts, mobile applications, and point of care websites. M-learning utilization will be shown by exploring usage data from a point of care website. This session will further explore advantages and disadvantages of microlearning platforms and methods of incorporating the platforms into more traditional learning methods. Broadening educator knowledge of microlearning tools will enable them to utilize a variety of teaching modalities and reinforce geriatrics curricula for learners. It also provides learners with resources to utilize independently when future knowledge gaps are identified.

